# Prevalence and Severity of Depression, Anxiety, and Stress Among COVID-19 Survivors in Urban Hazaribagh, Jharkhand: A Community-Based Cross-Sectional Study

**DOI:** 10.7759/cureus.109015

**Published:** 2026-05-17

**Authors:** Iqbal Farooqui, Mithilesh Kumar

**Affiliations:** 1 Community Medicine, Rajendra Institute of Medical Sciences, Ranchi, IND

**Keywords:** community-based study, depression anxiety stress, depression anxiety stress scales (dass-21), long-covid-19, psychological comorbidity

## Abstract

Background: Mental health sequelae following COVID-19 recovery are well-documented in large urban centers, yet community-based data from semi-urban India, particularly Jharkhand, remain scarce.

Objective: To assess the prevalence and severity of depression, anxiety, and stress among COVID-19 survivors in urban Hazaribagh using the Depression Anxiety Stress Scale-21 (DASS-21) and to identify associated sociodemographic and clinical factors.

Materials and methods: A community-based cross-sectional study was conducted from March 2022 to June 2022, following approval from the Institutional Ethics Committee (IEC), RIMS, Ranchi, obtained prior to data collection. Using systematic random sampling from Integrated Disease Surveillance Programme (IDSP) COVID-19 registers, every seventh from 1,065 first-wave cases and every 17th from 7,686 second-wave cases, 601 consenting recovered adults were enrolled from 640 contacted (response rate: 93.9%). The DASS-21 was administered in its validated Hindi version alongside a semi-structured questionnaire. Data were analyzed in JASP using chi-square and Fisher's exact tests; a value of p<0.05 was considered significant.

Results: Depression was the most prevalent domain: 337 (56.07%) scored above normal, with 154 (25.62%) moderate, 80 (13.31%) severe, and 44 (7.32%) extremely severe. Anxiety was present at any grade in 260 (43.26%), predominantly mild (211 [35.11%]); stress was elevated in 200 (33.28%). The wave of infection was significantly associated with all three domains (all p≤0.030). Hospitalization was strongly associated with anxiety and stress (both p<0.001). Age was significantly associated with anxiety (p<0.001) and stress (p=0.015); occupation with depression (p=0.045); and smoking with stress (p=0.014). Gender was not significantly associated with any domain.

Conclusion: COVID-19 survivors in urban Hazaribagh carry a substantial post-recovery psychological burden. Depression at moderate-to-extremely-severe intensity is the most predominant manifestation. Integrating routine DASS-21 screening into community-level post-COVID follow-up care, with priority for high-risk groups, is recommended.

## Introduction

The coronavirus disease 2019 (COVID-19) emerged as a major global public health emergency from December 2019 onwards. By mid-2022, more than 567 million infections had been noted, and 6.3 million deaths had been recorded globally. In India, approximately 43.8 million cases and 528,000 deaths were documented [1].

The psychological impact of COVID-19 infection persisted beyond the acute illness phase. Survivors frequently faced compounded stressors mainly due to mandatory quarantine, exposure to community mortality, economic disruption from lockdowns, unrelenting uncertainty about long-term health, and social stigma. Psychological resilience was further burdened by the unrestricted anxiety-amplifying information imparted through digital and social media [2]. A precedent from the past severe acute respiratory syndrome (SARS) epidemic noted in 2003 demonstrated that depression and post-traumatic stress disorder (PTSD) lasted in survivors for years after physical recovery [3].

Within India, systematic reviews have estimated pooled anxiety and depression prevalences of approximately 28-30% in the general population during the pandemic [4]. A global meta-analysis, Salari et al., also reported pooled prevalences of stress (29.6%), anxiety (31.9%), and depression (33.7%) across COVID-19-affected populations [5]. Survivor-specific data are considerably higher. Meena et al. documented depression and anxiety in 38.5% of 200 COVID-19 survivors at a tertiary hospital in Bhopal using the DASS-21 [6]. Chaudhary et al. similarly reported elevated rates among hospitalized COVID-19 patients and their families [7]. A systematic review of global evidence confirmed that COVID-19 survivors show significantly greater psychological morbidity than the non-infected general population [8]. Most published Indian studies originate from metropolitan tertiary hospitals, limiting generalizability to smaller cities with distinct sociodemographic profiles.

Hazaribagh, a municipal city in Jharkhand with an urban population of 237,994 (Census 2011) [9], recorded 1,065 COVID-19 cases during the first wave (June-September 2020) and 7,686 during the second wave (April-July 2021) per IDSP records. Its occupational profile, predominantly self-employed, housewives, and unemployed, represents a large segment of semi-urban India that remains almost entirely absent from post-COVID mental health research.

DASS-21, developed by Lovibond and Lovibond, is a validated 21-item self-report instrument assessing depression, anxiety, and stress across five severity levels [10]. It has demonstrated strong internal consistency [11] and is well-suited for community-based post-COVID screening. The aim of the present study was to assess the prevalence and severity of depression, anxiety, and stress among COVID-19 survivors in urban Hazaribagh using DASS-21 and to identify associated sociodemographic and clinical factors.

## Materials and methods

Study design and setting

A cross-sectional, community-based study was conducted in the urban area of Hazaribagh, Jharkhand, India, from March 2022 to June 2022.

Ethical clearance

The study was conducted after obtaining clearance from the Institutional Ethics Committee of RIMS, Ranchi, prior to the commencement of data collection. Written informed consent was obtained from all participants. Participant anonymity was maintained through numerical coding throughout.

Sample size

The sample size was calculated using the formula n = Z²pq/d². Meena et al. reported the prevalence of stress among COVID-19 survivors to be 11% [6], with Z=1.96 and d=5%, giving a minimum required sample of 151. Accounting for anticipated non-response, 640 individuals were contacted: 601 enrolled (response rate 93.9%).

Sampling technique

**Figure 1:**
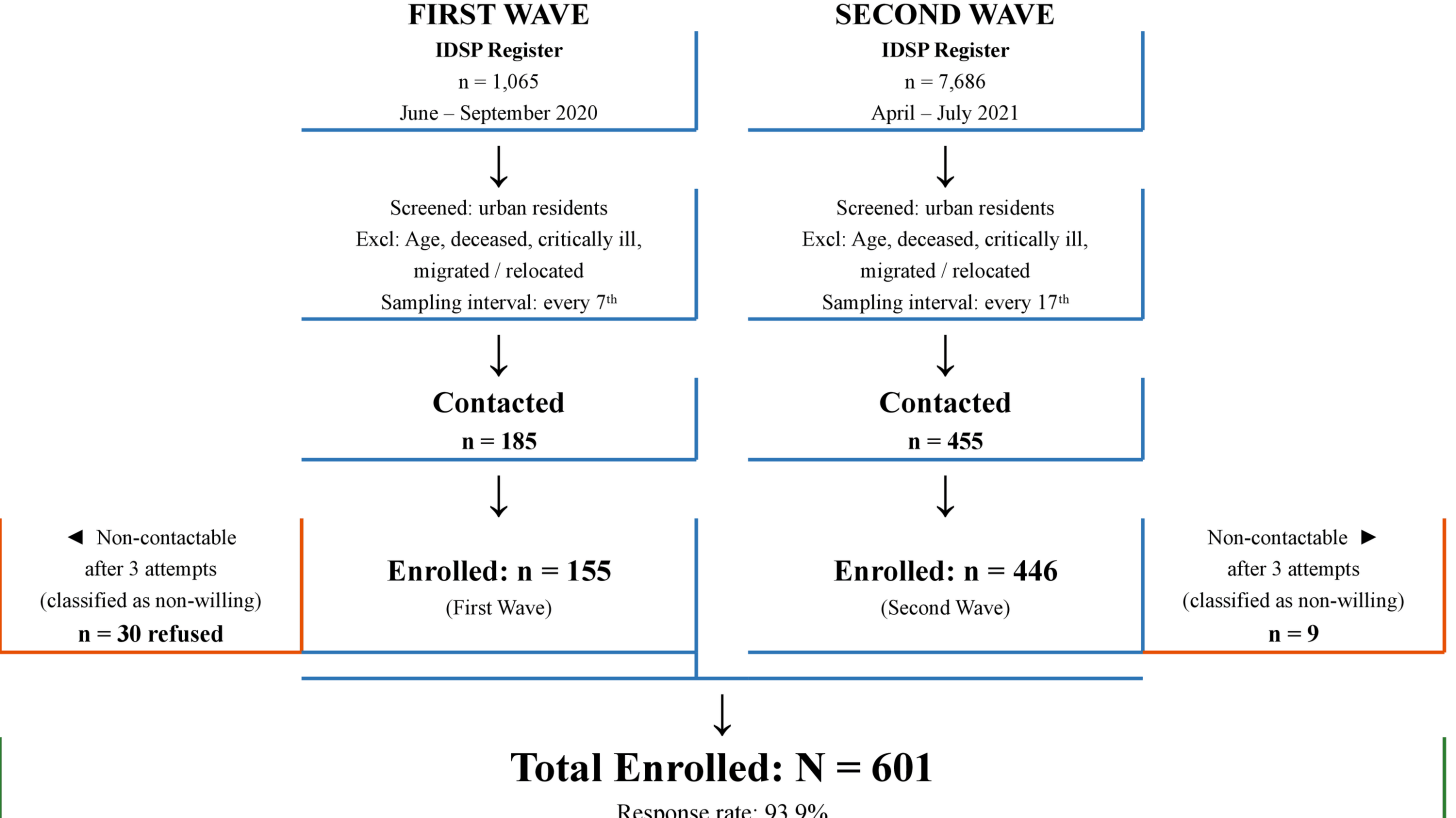
Participant flow diagram IDSP: Integrated Disease Surveillance Programme; DASS-21: Depression Anxiety Stress Scale-21. Data collection period: March–June 2022. The disproportionate first-to-second-wave enrollment ratio (~1:3) reflects the higher proportion of non-contactable temporary residents and migrant workers in the second-wave IDSP register.

The list of all COVID-19 positives was obtained from IDSP, Sadar Hospital, Hazaribagh, and systematic random sampling was applied following formal institutional permission. The register was further screened for urban residents, excluding deceased individuals, critically ill individuals, and those who had migrated or relocated. The study originally intended to enroll first-wave and second-wave participants in an approximately equal ratio. However, the second-wave IDSP register contained a substantially higher proportion of temporary residents and migrant workers relative to the first wave; many of these individuals could not be traced by the time of data collection or had relocated and were thus excluded per protocol. To account for this differential noncontactable proportion while maintaining systematic sampling, separate sampling fractions were derived: every seventh individual from the 1,065 first-wave cases (June-September 2020) and every seventeenth from the 7,686 second-wave cases (April-July 2021), yielding a final enrollment of 155 first-wave and 446 second-wave participants. Selected individuals were contacted by telephone; those unreachable after three separate contact attempts were classified as non-willing. The participant flow is illustrated in Figure 1.

Eligibility criteria

The inclusion and exclusion criteria applied in this study are presented in Table 1.

**Table 1:** Inclusion and exclusion criteria for participant selection DASS-21: Depression Anxiety Stress Scale-21; RT-PCR: Reverse transcription-polymerase chain reaction.

Inclusion Criteria	Exclusion Criteria
Laboratory-confirmed COVID-19 positive by RT-PCR or rapid antigen test during the first or second wave of the pandemic	Significant pre-existing medical illness capable of producing symptoms that overlap with DASS-21 scoring domains (including known psychiatric diagnoses, neurological disorders with affective manifestations, or active psychotropic medication use), thereby potentially confounding the psychological assessment
Aged 18 years or above	—
Willing to provide written or oral informed consent	—

Data collection instruments

A semi-structured questionnaire, pilot-tested on 30 rural Hazaribagh district residents and revised accordingly, collected: (i) sociodemographic data (age, gender, occupation, vaccination history); (ii) COVID-19 illness history (wave of infection, confirmation modality, hospitalization status, ward type); (iii) substance use history (smoking, non-smoked tobacco, alcohol); and (iv) DASS-21 [11]. All participants were interviewed over the telephone. The DASS-21 was administered in its validated Hindi version [12]. The DASS-21 comprises three seven-item subscales. Each raw subscale score is multiplied by two; severity is classified as normal, mild, moderate, severe, or extremely severe per established cut-offs [10].

DASS-21 use and permission

The DASS-21 is made freely available by the Psychology Foundation of Australia for non-commercial research and educational purposes without requiring a formal license or fee. Accordingly, no license application was required for this study.

Statistical analysis

Data were entered in Microsoft Excel and analyzed using JASP (version 0.16). Frequencies and percentages describe categorical variables. Associations between DASS-21 severity categories and categorical sociodemographic or clinical variables were assessed using Pearson's chi-square test; Fisher's exact test was applied where expected cell frequencies were below five. A two-tailed p-value <0.05 was considered statistically significant.

## Results

Sociodemographic and clinical profile

Of the 640 individuals contacted, 601 (93.9%) enrolled. Table 2 presents the full sociodemographic and clinical profile. The sample comprised 361 (60.07%) males and 240 (39.93%) females. The majority were aged 18-30 years (180 [29.95%]), self-employed (195 [32.45%]), and infected during the second wave (446 [74.21%]). At diagnosis, 571 (95.01%) were unvaccinated; by the interview date, 560 (93.18%) had since been fully vaccinated, 376 (66.43%) with Covishield and 190 (33.57%) with Covaxin. Hospitalization during acute illness was required by 76 (12.65%) participants, of whom 61 (80.26%) were admitted to a general ward, 12 (15.79%) to the intensive care unit (ICU), and 3 (3.95%) required ICU care with mechanical ventilation.

**Table 2:** Sociodemographic and clinical profile of COVID-19 survivors (N=601) ICU: Intensive care unit; COVID-19: Coronavirus disease 2019. Percentages are calculated as a proportion of the total sample (N=601).

Category	n (%)
Gender	
Male	361 (60.07%)
Female	240 (39.93%)
Age group (years)	
18–30	180 (29.95%)
31–40	157 (26.12%)
41–50	121 (20.13%)
51–60	79 (13.14%)
61–70	39 (6.49%)
>70	25 (4.16%)
Wave of infection	
First wave (June–September 2020)	155 (25.79%)
Second wave (April–July 2021)	446 (74.21%)
Occupation	
Self-employed	195 (32.45%)
Housewife	163 (27.12%)
Unemployed	128 (21.30%)
Service	112 (18.64%)
Labourer	3 (0.50%)
Vaccination status at time of COVID-19 diagnosis	
Unvaccinated	571 (95.01%)
Fully vaccinated	18 (3.00%)
Partially vaccinated	12 (2.00%)
Vaccine received (n=566)ᵃ	
Covishield	376 (66.43%)
Covaxin	190 (33.57%)
Substance use	
Smoking	125 (20.80%)
Tobacco — non-smoked forms	207 (34.44%)
Alcohol	116 (19.30%)
Hospitalization during acute illness	
Yes	76 (12.65%)
No	525 (87.35%)
Ward type (among hospitalized, n=76)	
General ward	61 (80.26%)
Intensive care unit (ICU)	12 (15.79%)
ICU with mechanical ventilation	3 (3.95%)

DASS-21 scores: overall distribution

Table 3 presents the DASS-21 severity distribution. Depression was the predominant psychological burden: 337 (56.07%) scored above the normal threshold, with a combined moderate-to-extremely-severe depression rate of 278 (46.26%), nearly one in two survivors. Anxiety at any grade was present in 260 (43.26%), predominantly mild (211 [35.11%]); only 49 (8.15%) reached the moderate-to-extremely-severe range. Stress was elevated in 200 (33.28%), with most in the moderate category (84 [13.98%]).

**Table 3:** DASS-21 severity distribution among COVID-19 survivors (N=601) DASS-21: Depression Anxiety Stress Scale-21. Values are n (%). Scores derived by multiplying raw subscale totals by two. Severity cut-offs per Lovibond and Lovibond (1995) [10]: Depression: Normal: 0–9, Mild: 10–13, Moderate: 14–20, Severe: 21–27, Extremely severe: ≥28. Anxiety: — Normal: 0–7, Mild: 8–9, Moderate: 10–14, Severe: 15–19, Extremely severe: ≥20. Stress: — Normal: 0–14, Mild: 15–18, Moderate: 19–25, Severe: 26–33, Extremely severe: ≥34.

Domain	Normal n(%)	Mild n(%)	Moderate n(%)	Severe n(%)	Extremely Severe n(%)	Total
Depression	264 (43.93)	59 (9.82)	154 (25.62)	80 (13.31)	44 (7.32)	601
Anxiety	341 (56.74)	211 (35.11)	17 (2.83)	16 (2.66)	16 (2.66)	601
Stress	401 (66.72)	51 (8.49)	84 (13.98)	47 (7.82)	18 (2.99)	601

DASS-21 scores by wave of infection

All three DASS-21 domains differed significantly between first- and second-wave survivors (Table 4). Depression was significantly higher proportionally among first-wave survivors, with 55 (35.5%) in the moderate range versus 99 (22.2%) in the second wave (χ²=16.900, df=4, p=0.002). For anxiety, first-wave survivors showed equal proportions in normal and mild categories (72 [46.5%] each), while second-wave survivors had a higher normal proportion (269 [60.3%]) (χ²=12.803, df=4, p=0.012). Stress also differed significantly, with first-wave survivors showing higher proportional moderate (31 [20.0%]) and severe stress (12 [7.7%]) compared to second-wave survivors (53 [11.9%] and 35 [7.8%], respectively) (χ²=10.708, df=4, p=0.030).

**Table 4:** DASS-21 severity scores stratified by wave of COVID-19 infection DASS-21: Depression Anxiety Stress Scale-21. *Statistically significant at p<0.05 (chi-square test, df=4). Percentages calculated within each wave subgroup. First wave: June–September 2020; Second wave: April–July 2021.

Domain	Wave	Normal n(%)	Mild n(%)	Moderate n(%)	Severe n(%)	Extremely Severe n(%)	p-value
Depression	First (n=155)	49 (31.6)	19 (12.3)	55 (35.5)	19 (12.3)	13 (8.4)	0.002*
	Second (n=446)	215 (48.2)	40 (9.0)	99 (22.2)	61 (13.7)	31 (6.9)	
Anxiety	First (n=155)	72 (46.5)	72 (46.5)	3 (1.9)	5 (3.2)	3 (1.9)	0.012*
	Second (n=446)	269 (60.3)	139 (31.2)	14 (3.1)	11 (2.5)	13 (2.9)	
Stress	First (n=155)	91 (58.7)	13 (8.4)	31 (20.0)	12 (7.7)	8 (5.2)	0.030*
	Second (n=446)	310 (69.5)	38 (8.5)	53 (11.9)	35 (7.8)	10 (2.2)	

Associations with sociodemographic and clinical variables

Table 5 summarizes chi-square statistics for all 14 variables across three DASS-21 domains. Age was significantly associated with anxiety (χ²=65.381, df=20, p<0.001) and stress (χ²=36.038, df=20, p=0.015); the >70-year group had the highest proportional extremely severe anxiety (5 [20.0%]) and severe stress (7 [28.0%]). Gender was not significantly associated with any domain (all p>0.05). Occupation was significantly associated with depression alone (χ²=26.721, df=16, p=0.045); unemployed participants had the highest proportion of extremely severe depression (11 [8.6%]). Vaccination status at diagnosis was significantly associated with anxiety only (χ²=24.132, df=8, p=0.002). Vaccine type was significantly associated with anxiety (χ²=14.434, df=4, p=0.006) and stress (χ²=12.684, df=4, p=0.013). Smoking was significantly associated with stress (χ²=12.574, df=4, p=0.014): smokers showed proportionally higher moderate (19 [15.2%]) and severe stress (15 [12.0%]) than non-smokers. hospitalization was strongly associated with anxiety (χ²=59.727, df=4, p<0.001) and stress (χ²=25.851, df=4, p<0.001), but not depression (p=0.140). Duration of admission was additionally significant for anxiety (χ²=30.361, df=16, p=0.016) and stress (χ²=31.901, df=16, p=0.010).

**Table 5:** Chi-square (χ²) associations of DASS-21 domains with sociodemographic and clinical variables DASS-21: Depression Anxiety Stress Scale-21; NS: Not significant (p≥0.05); df: Degrees of freedom. *Significant at p<0.05 (two-tailed). Fisher's exact test is applied where expected cell frequency is <5.

Variable	Depression χ²(p)	Anxiety χ²(p)	Stress χ²(p)	df	Significant Domain(s)
Age group	14.105 (0.825)	65.381 (<0.001)	36.038 (0.015)	20	Anxiety, Stress
Gender	2.268 (0.687)	4.349 (0.361)	5.066 (0.281)	4	NS
Wave of infection	16.900 (0.002)	12.803 (0.012)	10.708 (0.030)	4	All three
Number of COVID-19 episodes	7.176 (0.127)	1.220 (0.875)	2.515 (0.642)	4	NS
Occupation	26.721 (0.045)	17.401 (0.360)	18.393 (0.301)	16	Depression
Vaccination at diagnosis	5.940 (0.654)	24.132 (0.002)	14.652 (0.066)	8	Anxiety
Vaccine type received	7.384 (0.117)	14.434 (0.006)	12.684 (0.013)	4	Anxiety, Stress
Current vaccination status	12.102 (0.147)	13.485 (0.096)	9.373 (0.312)	8	NS
Smoking	1.452 (0.835)	3.850 (0.427)	12.574 (0.014)	4	Stress
Tobacco use (non-smoked)	2.193 (0.700)	8.730 (0.068)	4.135 (0.388)	4	NS
Alcohol use	5.196 (0.268)	8.455 (0.076)	5.075 (0.280)	4	NS
Hospitalization	6.931 (0.140)	59.727 (<0.001)	25.851 (<0.001)	4	Anxiety, Stress
Ward type	7.594 (0.474)	12.396 (0.134)	8.517 (0.385)	8	NS
Duration of admission	14.567 (0.557)	30.361 (0.016)	31.901 (0.010)	16	Anxiety, Stress

Among hospitalized participants, 5 (6.6%) had extremely severe anxiety, and 9 (11.8%) had severe anxiety, versus 11 (2.1%) and 7 (1.3%), respectively, among non-hospitalized participants. For stress, 11 (14.5%) hospitalized participants were in the severe range versus 36 (6.9%) among non-hospitalized. Depression distributions did not differ significantly (p=0.140) (Table 6).

**Table 6:** DASS-21 scores stratified by hospitalization status during acute COVID-19 illness DASS-21: Depression Anxiety Stress Scale-21; NS: Not significant; ICU: intensive care unit. *Statistically significant at p<0.001 (chi-square test, df=4). Values are n (%). Chi-square values reported as χ²(df=4).

Hospitalization Status	Normal n(%)	Mild n(%)	Moderate n(%)	Severe n(%)	Extremely Severe n(%)	χ²(p-value)
Anxiety – Hospitalized (n=76)	25 (32.9)	29 (38.2)	8 (10.5)	9 (11.8)	5 (6.6)	59.73 (<0.001)*
Anxiety – Not hospitalized (n=525)	316 (60.2)	182 (34.7)	9 (1.7)	7 (1.3)	11 (2.1)	
Stress – Hospitalized (n=76)	32 (42.1)	9 (11.8)	21 (27.6)	11 (14.5)	3 (3.9)	25.85 (<0.001)*
Stress – Not hospitalized (n=525)	369 (70.3)	42 (8.0)	63 (12.0)	36 (6.9)	15 (2.9)	
Depression – Hospitalized (n=76)	25 (32.9)	12 (15.8)	21 (27.6)	10 (13.2)	8 (10.5)	6.93 (0.140) NS
Depression – Not hospitalized (n=525)	239 (45.5)	47 (9.0)	133 (25.3)	70 (13.3)	36 (6.9)	

## Discussion

This study provides the first community-based estimates of post-COVID psychological morbidity from Hazaribagh, Jharkhand. Depression (337 [56.07%]), anxiety (260 [43.26%]), and stress (200 [33.28%]) were all prevalent, with depression emerging as the dominant and most severe manifestation. A total of 278 (46.26%) of the cohort fell in the moderate-to-extremely-severe range.

The depression prevalence substantially exceeds that reported by Meena et al. (38.5%) among COVID-19 survivors at a tertiary hospital in Bhopal using the same instrument [6]. The systematic review by Sharma et al. [4] reported a pooled depression of approximately 30% in the general Indian population during the pandemic, figures considerably lower than our survivor-specific community estimate. The global meta-analysis by Salari et al. reported a pooled depression of 33.7% across COVID-19-affected populations [5], still below our survivor cohort, further highlighting the elevated burden in those who directly experienced the illness. Several factors likely account for this gap: our community-based sampling portrays a broader illness-severity spectrum; 446 (74.21%) of our cohort survived the more traumatic second wave; and the study was conducted months after the respective wave peaks, during which unresolved grief, occupational disruption, and reduced pandemic-era social solidarity may have consolidated depressive pathology.

The predominance of mild anxiety (211 [35.11%]) with relatively few participants reaching moderate-to-extremely-severe levels (49 [8.15%]) is consistent with the natural history of pandemic-related psychological distress, acute anticipatory fear attenuating post-recovery while chronic depressive symptoms persist. This temporal shift has been described in post-SARS mental health literature [3]. A systematic review by Vindegaard and Benros confirmed that COVID-19 survivors consistently show elevated depression and PTSD rates compared to the non-infected general population [8], supporting the direction of our findings.

The significant association of the wave of infection with all three psychological domains is a notable finding. First-wave survivors showed proportionally higher moderate depression and stress, potentially reflecting greater early-pandemic stigma and community isolation. Second-wave survivors contributed more absolutely severe and extremely severe cases, consistent with the greater clinical severity of the delta variant surge and widespread community mortality in 2021 [13].

Occupational gradients in depression underscore the socioeconomic dimensions of post-COVID psychological vulnerability. Unemployed participants had the highest extremely severe depression rate (11 [8.6%]), consistent with evidence that economic insecurity compounds the psychological toll of serious illness [14]. Hospitalization was the strongest individual clinical predictor of anxiety and stress, consistent with evidence that inpatient infectious disease isolation creates enduring anxiety templates [15]. The absence of a significant gender effect diverges from global psychiatric epidemiology [16] and may reflect social desirability effects in self-reporting, the male predominance of our sample, or equalization of psychological vulnerability under the shared experience of potentially fatal illness.

The associations of vaccine type and vaccination status at diagnosis with DASS-21 anxiety and stress scores should be interpreted with caution, as these variables are likely confounded by calendar time, wave of infection, and health-seeking behavior. No direct causal relationship should be inferred from these cross-sectional bivariate findings.

Limitations

Limitations include the cross-sectional design (precluding causal inference); absence of a pre-COVID psychological baseline and a non-COVID control group; possible underrepresentation of informally tested individuals; social desirability bias in telephonic self-reporting; confounding by differential time since infection across waves; and restriction to the urban area of a single city in Jharkhand. Findings may not generalize to rural or tribal populations in the region.

## Conclusions

COVID-19 survivors carry a substantial and clinically significant post-recovery psychological burden. Depression, predominantly at moderate-to-extremely-severe intensity, is the most predominant manifestation, followed by anxiety and stress. Hospitalization, advancing age, wave of infection, unemployment, and smoking are the principal risk correlates identified. These findings highlight the need for integrating routine post-COVID psychological screening using the DASS-21 into community-level follow-up care at district hospitals and primary health centers, with priority given to high-risk groups, including hospitalized survivors, the elderly, and the economically vulnerable. Mental health referral pathways should be activated within the existing public health infrastructure for those scoring above clinical thresholds.

## Appendices

Declaration on the use of artificial intelligence writing assistance

In accordance with ICMJE recommendations and Cureus editorial policies, the authors declare that AI writing assistance (Claude, Anthropic) was used in the drafting, editing, and language refinement of this manuscript. All study data, statistical analyses, intellectual content, scientific interpretation, and conclusions are entirely the authors' own work. The AI tool was not involved in study design, data collection, data analysis, or the generation of any results presented herein. All authors have duly reviewed the final manuscript in full, accept complete responsibility for its accuracy and integrity, and approve this submission.
